# Medical Department of Niagara University

**Published:** 1888-07

**Authors:** 


### *MEDICAL DEPARTMENT OF NIAGARA UNIVERSITY.*

By the announcement just published, we learn that the next course of lectures is to begin on the evening of September 19th with an introductory address by Professor H. D. Ingraham, and will close April 9, 1889. Some changes have taken place in the personnel of the faculty, which are worthy of note. In the first place, death has removed from it Professor A. R. Davidson, one of its most valued members, and thus inflicted a loss which is most severe. Professor Wheeler, who has served the school temporarily, but most acceptably, during his stay here as United States Marine Surgeon, is soon to be transferred to another port, and his name disappears from the list of teachers. Professor Daniels and Dr. Edward Clark, who have been loyal and earnest workers in the school since its organization, have felt that their own interests would best be subserved by withdrawing, and have resigned. The vacancies thus caused in the departments of surgery, anatomy, and medical chemistry have been filled as follows: Dr. Herman Mynter, formerly one of the editors of this JOURNAL, has been appointed Professor of Surgery and Clinical Surgery; Dr. Herbert Mickle, heretofore Lecturer on Pathology, Professor of Descriptive and Surgical Anatomy; and Dr. John A. Miller, of this city, Lecturer on Medical Chemistry and Toxicology.

Each of these appointees has much to recommend him for the respective position to which he has been elected. Dr. Mynter comes to the chair of Surgery in the prime of life and full vigor of health, with a fine education and with a high reputation as a surgeon. He has had a large surgical experience, both in private and hospital practice, and for several years has given lectures on clinical surgery, which have been considered of a high order. He is a man of great energy and zeal, and will, without doubt, prove an instructive and interesting teacher. In entering the school, he also becomes surgeon to the Sisters of Charity Hospital, which he hopes to make most complete and successful in its surgical department, and a center of attraction throughout the city and surrounding country for cases requiring surgical treatment, as well as for students and physicians who desire to witness important surgical work.

Dr. Mickle takes the chair of Anatomy with a thorough preparation and knowledge of his subject. He is a man of excellent education, and has already, in his connection with the school from its beginning, proved himself a most interesting and valuable teacher. His study in the hospitals of London, his work here, both privately and in our hospitals, his experience as a didactic and clinical teacher, especially recommend him to the promotion which he has now received. It is safe to predict that he has a bright future before him; and we shall be glad to see him no less distinguished than his brother, Dr. Wm. Julius Mickle, of London, whose fame as an alienist is world-wide.

Dr. John A. Miller, who has been appointed Lecturer on Medical Chemistry and Toxicology, although less known than Dr. Mynter or Dr. Mickle, is a man of excellent training and ability, and will be heard of in the future. He is a resident of this city, although he has been absent for several years equipping himself as a professional chemist. During the past three years, he has been studying at the University of Berlin, and soon returns crowned with the honors of the institution. He comes to Buffalo to devote himself wholly to chemical work, and to enter a field which is fertile and comparatively unoccupied. The school is fortunate in adding one of his qualificatious to its teaching body, and thus, in a measure at least, compensating for the loss caused by Dr. Davidson's death.

Some other changes have been made in the auxiliary faculty, and here, too, the members are all able and worthy men. On the whole, the additions to the medical faculty are commendable and are sources of congratulation.

